# Bioactive Thiazine and Benzothiazine Derivatives: Green Synthesis Methods and Their Medicinal Importance

**DOI:** 10.3390/molecules21081054

**Published:** 2016-08-15

**Authors:** Syed Lal Badshah, Abdul Naeem

**Affiliations:** 1 National Center of Excellence in Physical Chemistry, Peshawar University, Peshawar, Khyber Pukhtoonkhwa 25120, Pakistan; 2 Department of Biochemistry, Abdul Wali Khan University Mardan, Khyber Pukhtoonkhwa 25120, Pakistan; 3 Department of Chemistry, Islamia College University Peshawar, Peshawar, Khyber Pukhtoonkhwa 25120, Pakistan

**Keywords:** thiazine, green synthesis, biologically active, antibacterial activity, anticancer agents

## Abstract

Thiazines are a group of heterocyclic organic compounds that are still largely unexplored for their pharmacological activities. There are different available methods for the synthesis of thiazine derivatives in the literature. In this review, we discuss available methods of thiazine preparation through green synthesis methods. Beside their synthesis, many thiazine derivatives are biologically active and play an important role in the treatment of various diseases and show promising results of varying degrees, where they act as antibacterial, antifungal, antitumor, antimalarial, antineoplastic, antiviral, anti-inflammatory, analgesic and anticancer agents and thus they represent an interesting class of heterocyclic medicinal compounds worthy of further exploration.

## 1. Introduction

**Figure 1:**
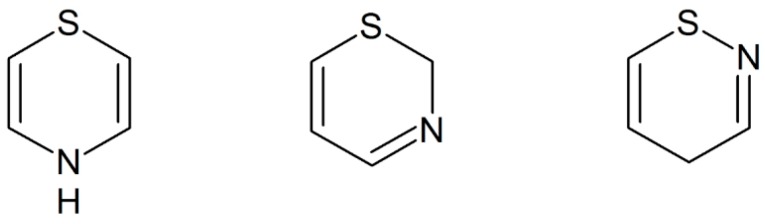
Structures of 1,4-, 1,3- and 1,2-thiazine.

Heterocyclic chemistry research encompasses almost half of the organic chemistry research throughout the world. A huge amount of bioactive organic compounds that contain heterocyclic frameworks play a vital part in the medicinal field. It is commonly reported that heterocycles having sulphur or nitrogen atoms or both of them are the general features present in the structures of most of the pharmaceutical and natural compounds [1,2]. They also act as multidentate ligands for different metals due to the presence of nitrogen and sulfur atoms and are thus used extensively in coordination chemistry to obtain new frameworks with potential bioactivity [2]. According to Joshi et al., it has also been identified that several heterocyclic compounds in the developmental phase have the potential to be part of new drugs and also play an important role in modern drug discovery [3]. Heterocyclic thiazine derivatives are important because they are biological constituents of many biomolecules and drugs [3]. The thiazines possess a nitrogen and sulphur atoms in a six member ring (Figure 1), that is believed to be important for their antifungal, anticonvulsant, and antiviral activities. The uniqueness and resourcefulness of the simple thiazine chemical structure and easy availability make thiazines and their derivatives amongst the most gifted sources of bioactive compounds [4,5]. There are a few review articles [6,7,8,9] available on thiazine compounds, but they are either focused mostly on 1,3-thiazines or lack comprehensiveness and there was a perceived need to discuss current green synthesis methods and their biological importance, with the aim of showing the importance of the data published to date on the synthesis and biological activities of different thiazines and their analogues. The present work clearly establishes the importance of thiazine and its derivatives for the synthesis of a wide variety of heterocyclic compounds through environmentally friendly methods that are of academic and pharmaceutical industry interest [4]. Moreover, during these syntheses the yield of the desired compounds is often high and they may be obtained in a single step. It has been noticed that structural modifications in the thiazine and its derivatives result in valuable medicinal properties that should be further explored through structure activity relationship (SAR) methods for the development of highly potent compounds against multi-drug resistant microorganisms and other diseases. Thus, the research to explore different avenues of chemical modifications of thiazine and its available derivatives to obtain novel active compounds should be continued. Therefore, it is of utmost timeliness to briefly review the novel green, synthetic methodologies and biological activities of thiazine and their derivatives.

The current collected data for the preparation and biochemical applications of thiazines and their derivatives relate to the green approach, i.e., reactions using microwave irradiation, sonication, grinding techniques, solvent-free conditions, nanoparticles, ionic liquids, etc. are presented in this review. The reagents used for the synthesis are ferric chloride, cesium carbonates, tetraphenyl phosphine, thiourea, ptolylsulphonic acid, palladium catalyst, perchloric acid, phosphomolybdic acid, 1-benzyl-3-methyl imidazolium hydrogen sulphate, etc. [9].

## 2. Green Syntheses of Thiazines and their Derivatives

**Scheme 1:**
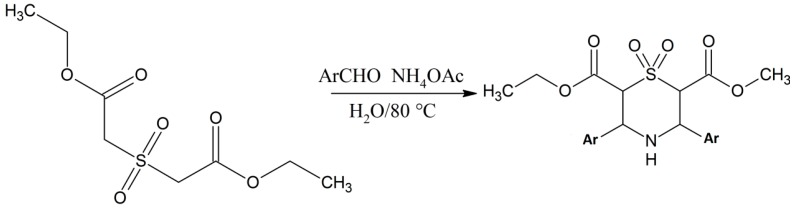
Eco-compatible preparation of the 2,6-dicarbethoxy-3,5-diphenylltetrahydro-1,4-thiazine-1,1-dioxides [10].

Edayadulla and Ramesh [10] synthesized a derivative of 1,4-thiazine, namely 2,6-dicarbethoxy-3,5-diphenyltetrahydro-1,4-thiazine-1,1-dioxide by taking diethyl 2,2-sulfonyldiacetate and benzaldehyde in water, to which ammonium acetate was added at room temperature [10]. The reactants were gently stirred for 3 h at 80 °C and after that they were cooled down to room temperature, and the product was extracted with ethyl acetate. The upper organic layer was dehydrated completely using the anhydrous Na_2_SO_4_ and concentrated at reduced pressure to obtain the crude product, which was purified and recrystallized using ethanol as a solvent to give the pure target compound in excellent yield [10]. The chemical structure of the compound was determined by different spectroscopic and analytical techniques (Scheme 1) [10]. Test results of these compounds on mice models showed that they effective anticonvulsants [10].

**Scheme 2:**
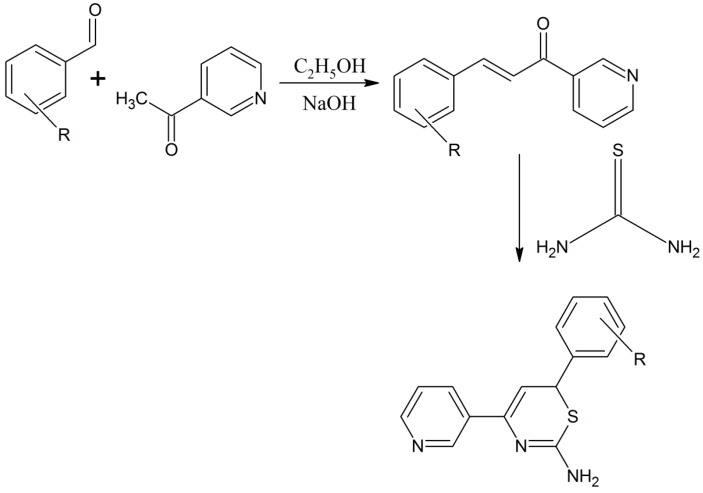
Formation of azachalcone compound from acetylated pyridine and benzaldehyde and its subsequent use for the synthesis of a thiazine derivative; R represents m-NO_2_, p-OH, m-Br, m-OCH_3_, p-N(CH_3_)2 [11].

Kadhim reported a novel azachalcone compound that was prepared through the reaction of acetylated pyridine with 4-hydroxybenzaldehyde [11]. Later the azachalcone was treated with thiourea to give a high yield of the thiazine derivative ((*E*)-3-(4-hydroxyphenyl)-1-(pyridin-3-yl) prop-2-en-1-one) (Scheme 2) [11]. The synthesized compound was characterized by infrared, ultra-violet, ^1^H-NMR spectroscopy and elemental analysis. The obtained compound were tested for anti-bacterial and anti-fungal properties and showed good activities (Scheme 2) [11].

**Scheme 3:**
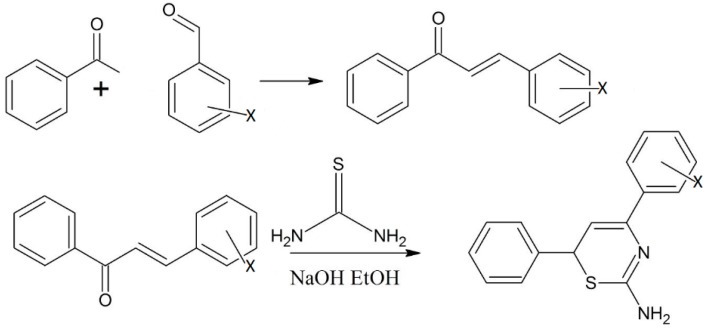
Preparation of thiazine derivatives from benzaldehyde and acetophenone through chalcones. Where X = *o*-OH; *p*-N(CH_3_)_2_; *m*-OCH_3_ [12].

According to Elarfi et al., chalcone derivatives can be synthesized by reacting benzaldehyde derivatives with acetophenone [12]. In the second step of this reaction, the product obtained was allowed to react with thiourea to form thiazines and other related compounds. The products obtained were characterized by elemental analysis, electromagnetic induction spectroscopy (EMIS), proton NMR and infrared spectroscopy [12]. The thiazine and its related derivatives were checked for bioactivity using some antibacterial tests (Scheme 3) [12].

**Scheme 4:**
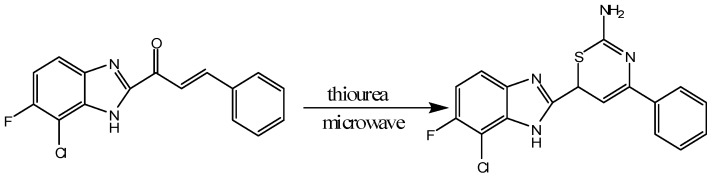
Synthesis of 1,3-thiazine derivatives under microwave irradiation [13].

Gayathri et al. reported that thiazine derivatives containing fluorine, chlorine and benzimidazole can be synthesized by microwave induced reactions [13]. They took 1-(7-chloro-6-fluoro-1*H*-benzo[*d*]imidazol-2-yl)-3-arylprop-2-en-1-one and cyclized it with thiourea in a microwave induced reaction producing the 1,3-thiazine derivative 6-(7-chloro-6-fluoro-1*H*-benzo-2-yl)-4-phenyl-6*H*-1,3-thiazine-2-amine [13]. The synthesized compounds were confirmed and characterized by thin layer chromatography (TLC), EMIS, elemental analysis, proton NMR and infrared spectroscopy. The manufactured compounds were screened for analgesic and antibacterial activities showing potent activities in both areas (Scheme 4) [13].

**Scheme 5:**
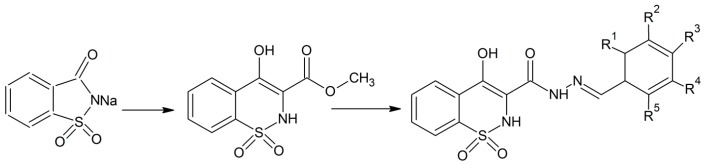
General Scheme for the synthesis of different *N*-[(1*E*)-arylmethylidene]-4-hydroxy 2*H*-1,2-benzothiazine-3-carbohydrazide 1,1-dioxides derivatives [14].

Zia-ur-Rehman et al. synthesized a series of analogues of 4-hydroxy-*N-*(benzylidene)-2*H*-benzo [*e*][1,2]thiazine-3 carbohydrazide 1,1-dioxides [14]. They performed *N*-alkylaton of sodium saccharin with methyl chloroacetate in the presence of ultrasonic irradiation [14]. Both the reagents were dissolved in dimethyformamide for the ultrasonic irradiation. The advantage of this process is that it can be done at lower temperatures and in a short period of time. In the next stage, the five membered isothiazole ring was converted into the thiazine ring under inert atmosphere conditions and then treated with hydrazine which was followed by reaction with benzaldehydes to give the desired products in high yield (Scheme 5) [14]. Some of the derivatives have good antibacterial properties, while most of the products possess antioxidant activities. Recently the same research group has expanded the synthesis of these 1,2-benzothiazine-3-carbohydrazide 1,1-dioxides using a microwave-assisted method that gives the products in high yield [15]. Zia-ur-Rehman et al. have also performed crystallographic studies of the 1,2-benzothiazine compounds to get better information about their molecular structure in terms of bond lengths, bond angles and various possible conformational states [16].

**Scheme 6:**
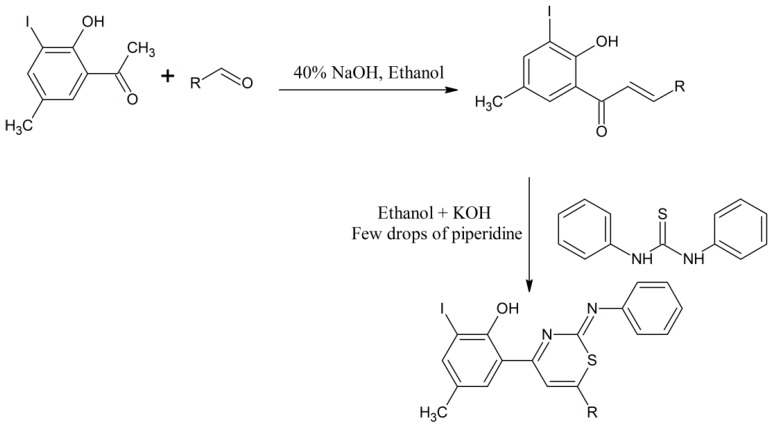
Formation of chalcones and their cyclization with diphenylthiourea in ethanol using KOH and few drops of piperidine as a catalyst [17].

Dighade et al. synthesized different chalcones through a condensation process of 2-hydroxy-3-iodo-5-methylacetophenone as a starting material with different aromatic aldehydes in ethanol using 40% sodium hydroxide solution as a catalyst [17]. The chalcones produced were cyclized with diphenylthiourea in ethanol using KOH and a few drops of piperidine as a catalyst. All the thiazine compounds were confirmed and categorized by elemental analysis, EIMS, ^1^H-NMR and infrared spectroscopies (Scheme 6) [17].

**Scheme 7:**
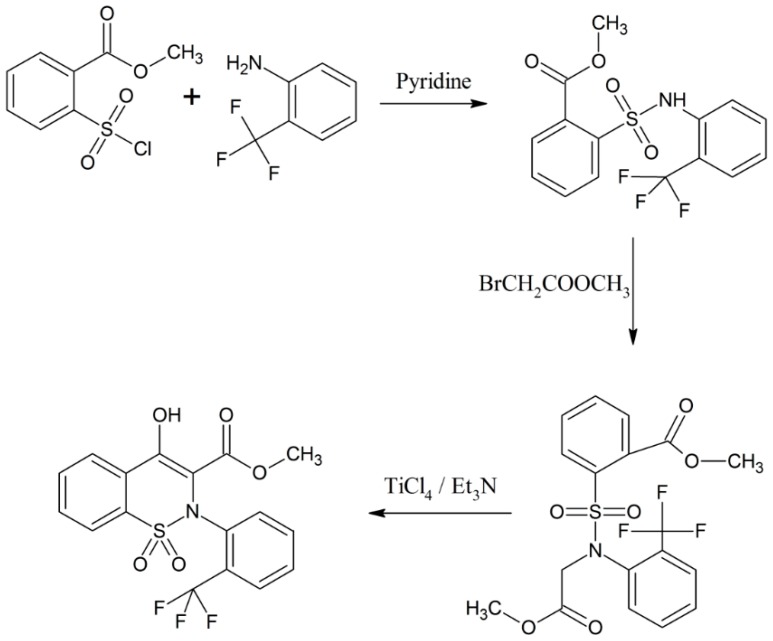
Formation of 1,2-benzothiazine derivatives using methyl-2-(chlorosulfonyl) benzoate and 2-(trifluoromethyl)aniline [18].

Bunker and colleagues synthesized and characterized a group of 1,2-benzothiazine analogues [18]. Methyl-2-(chlorosulfonyl)benzoate and 2-(trifluoromethyl)aniline were taken as starting materials and the whole benzothiazine synthesis was completed in three steps [18]. It was claimed that these 1,2-benzothiazine derivatives are useful in treating hypertension and as endothelial antagonists (Scheme 7) [18].

**Scheme 8:**
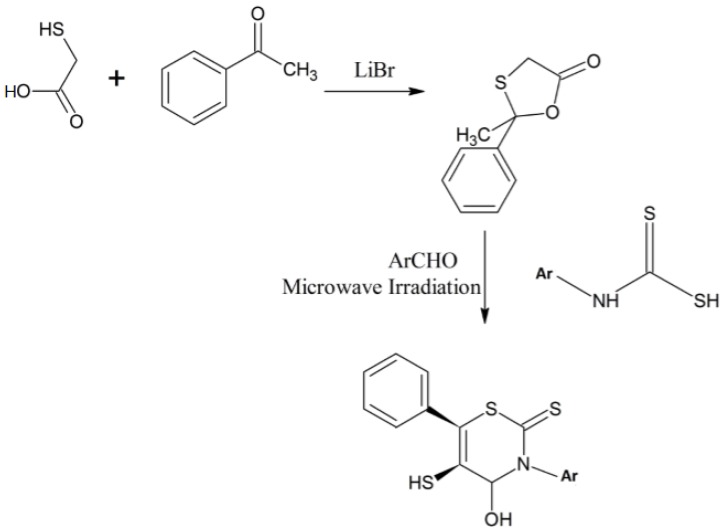
Three-component expeditious method for the synthesis of 1,3-thiazine derivatives [19].

Yadav et al. developed a new, expeditious, three-component method for the synthesis of 3,6-diaryl-5-mercaptoperhydro-2-thioxo-1,3-thiazin-5-ones 1,3-thiazine analogues from 2-methyl-2-phenyl-1,3-oxathiolan-5-one under solvent-free conditions [19]. They synthesized 2-methyl-2-phenyl-1,3-oxathiolan-5-one [20] from 2-mercaptoacetic acid and acetophenone using lithium bromide as a catalyst [19]. This intermediate was treated with a benzaldehyde and *N*-aryldithio carbamic acid to synthesize the desired product in a one-pot synthesis method using microwaves [19]. It is diastereoselective process and involves tandem Knoevenagel, Michael and ring transformation steps, as presented in Scheme 8 [19,20].

Torres-García et al. reported zinc complexes of a new ligand 2-(3,5-diphenyl-1-pyrazolyl)-1,3-thiazine (DPhPzTz) [21]. They treated a methanol solution of DPhPzTz with a methanol solution of zinc chloride [21]. After completion of the reaction and gentle dehydration of the solution at room temperature, colorless crystal of the complex ZnCl_2_(DPhPzTz) were obtained [21]. The product obtained was confirmed and characterized through elemental analysis and spectroscopic techniques, while the crystal structure was also determined. In addition, Torres-García and coworkers investigated the phagocytosis process of human neutrophils that was increased when treated with the Zn (II) complexes [21] and similar observations were reported for cadmium complexes with other ligands [22].

**Scheme 9:**
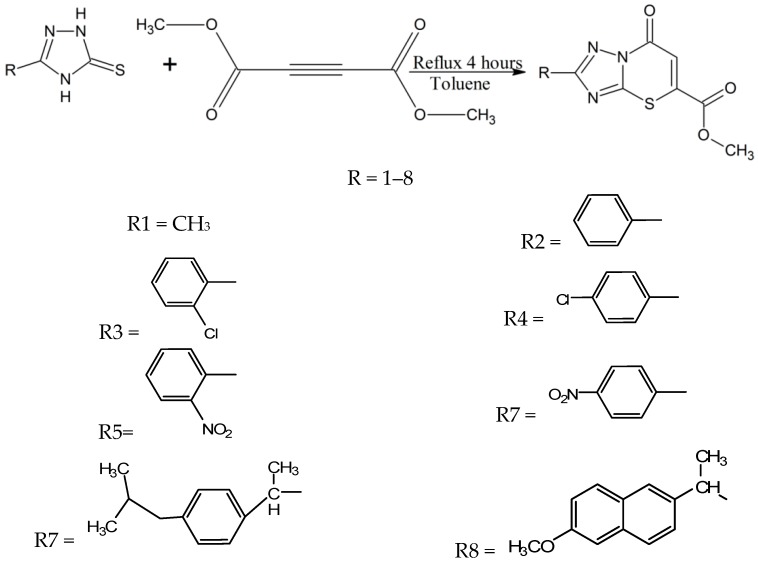
Synthesis of thiazine derivatives using 1,2,4-triazole-5-thiones as a starting material [23].

Tozkoparan and coworkers synthesized a series of 5-carbomethoxy-2-substituted-7*H*-1,2,4-triazolo(3,2-*b*)-1,3-thiazine-7-one 1,3-thiazine derivatives [23]. According to their reaction (Scheme 9), these compounds can be made by reacting 1,2,4-triazole-5-thiones with dimethyl acetylene dicarboxylate using toluene as a solvent under reflux conditions. They purified and characterized their thiazine compounds by different spectroscopic methods and elemental analysis. The synthesized thiazine analogues exhibit analgesic, anti-inflammatory activities and other interesting characteristics [23]. Most of the derivatives showed remarkable anti-inflammatory activity against edema caused by serotonin, carrageenan and in the inhibition of diarrhea tests [23], thus these novel 1,3-thiazines could be used in the future for the treatment of inflammatory pain after further laboratory trials. These compounds were also found safe for gastric ulcer treatment [23].

**Scheme 10:**
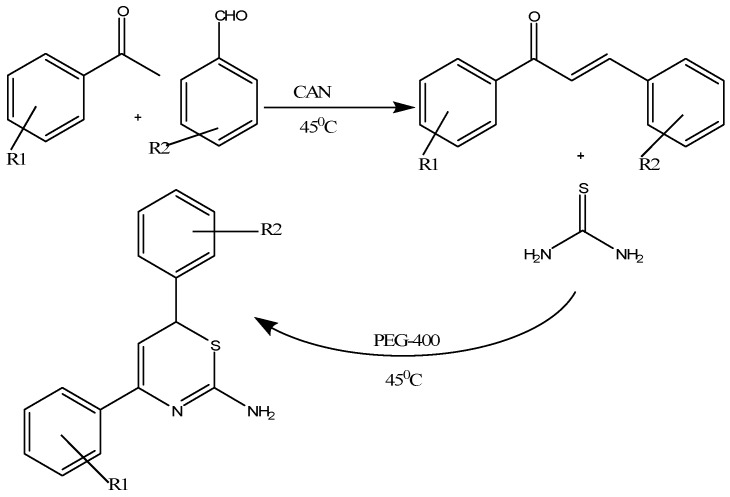
Ceric ammonium nitrate catalyzed preparation of 1,3-thiazines in polyethylene glycol [24].

Singh and colleagues developed an innovative, easy to use, inexpensive and highly efficient one pot, multi-component ceric ammonium nitrate (CAN)-catalyzed preparation of 1,3-thiazine derivatives in polyethylene glycol (PEG) 400 by mixing substituted acetophenones, aromatic aldehydes and thiourea in PEG-400 in specific amounts [24]. The reaction mixture was gently stirred while maintaining a uniform temperature of 45 °C until the formation of product was confirmed through TLC (Scheme 10). The desired 1,3-thiazines were isolated through a mixture of ether and PEG-400 that forms two separate layers as PEG is insoluble in ether and their structures were confirmed by elemental analysis and spectroscopic analysis [24]. These thiazine derivatives act as potent inhibitors of Gram-negative bacteria by targeting 4-diphosphocytidyl-2-*C*-methyl-d-erythritol (IspE) kinase [24].

**Scheme 11:**
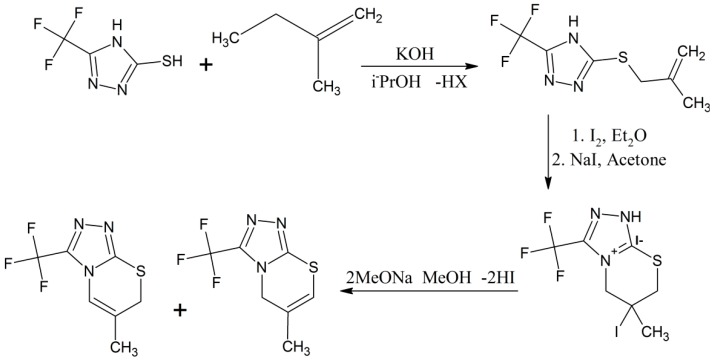
Alkenation reaction for the synthesis of triazole-based thiazine heterocyclic compounds [25].

Il’inykh and coworkers made *S*-alkenyl forms of 5-(trifluoromethyl)-4*H*-1,2,4-triazole-3-thiol compounds by treating various alkenyl halides with 2,4-triazoles conjugated with trifluoromethyl and thiol groups [25]. Furthermore the chemical process between the *S*-alkenyl derivatives and iodine molecules occurs regiospecifically producing new fused halogenated (1,2,4)triazole(3,4-*b*)(1,3)thiazine compounds (Scheme 11) [25]. The chemical structures of these products were confirmed by elemental analysis, IR and mass spectroscopy and different isotopic (^1^H-, ^13^C- and ^19^F-) NMR spectroscopies, including state of the art 2D ^1^H-^13^C-HSQC, ^1^H-^1^H-COSY, ^1^H-^13^C-HMBC correlations, and also by single-crystal XRD technique for better results [25].

**Scheme 12:**
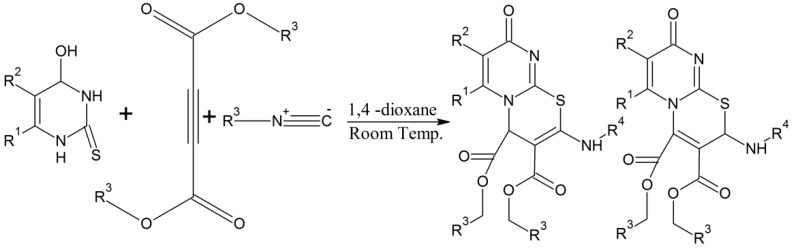
Chemical preparation of pyrimido (2,1-*b*)(1,3)thiazine derivatives [26].

According to Baharfar et al. pyrimido(2,1-*b*)(1,3)thiazine derivatives can be synthesized initially by the combination of an isocyanide and a dialkyl acetylenedicarboxylate that forms an active zwitterionic intermediate. This zwitterionic form of the intermediate is treated with thiouracil that forms ketenimine as an intermediate. This ketenimine undergoes chemical cyclization and rearrangement to form the desired product in high yield (Scheme 12) [26]. During the course of the reaction, the contents are stirred for a day at 25 °C. Baharfar and colleagues observed the formation of intermediates by analyzing them on TLC, and after completion of the whole process, the solvent was removed and the thiazines were purified by silica gel column chromatography using *n*-hexane and ethyl acetate as a solvents in a three to one volumetric ratio [26].

**Scheme 13:**
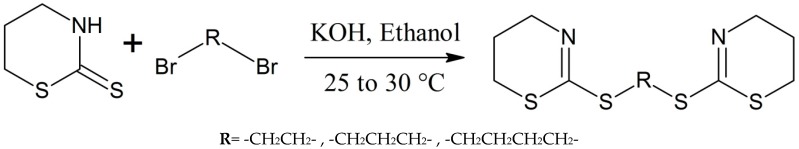
Formation of multithioether derivatives of thiazine [27].

Wang et al. made a series of novel multithioether derivatives using the reaction of thiazinethiones with alkyl dibromides following Scheme 13 [27]. The chemical properties and structures of these thiazine analogues were studied through EIMS, elemental analysis, IR, and ^1^H-NMR spectroscopy. The synthesized compounds were tested for antitumor activity and showed good results [27].

**Scheme 14:**
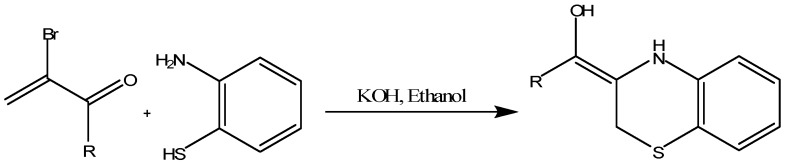
Synthesis of 1,4-thiazine derivatives from α-bromopropenone derivative and 2-amino-thiophenol in ethanolic potassium hydroxide solution [28].

El Shehry et al. reported that 1,4-thiazine derivatives can be synthesized by the reaction of α-bromopropenone derivatives with 2-aminothiophenol in ethanolic solution of potassium hydroxide (Scheme 14) [28]. These thiazine derivatives were tested for antimolluscicidal activities and the results showed that they exhibited moderate inhibitory power [28].

**Scheme 15:**
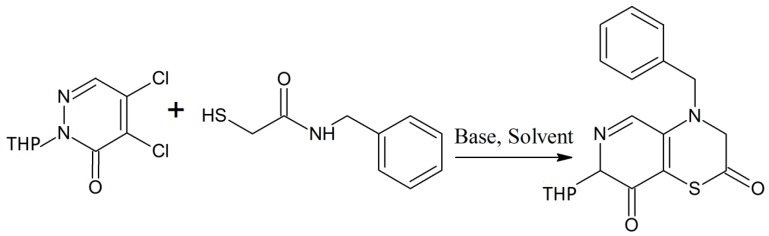
One-pot synthesis of pyridazino[4,5-*b*][1,4]thiazine-diones involving a Smiles rearrangement process [29].

Zhao et al. described a resourceful one-pot cyclization reaction process for the preparation of pyridazino(4,5-*b*)(1,4)thiazine-dione analogues following the famous Smiles rearrangement method [29]. They obtained a number of ideal compounds in high yields without any side reaction products. Moreover, this is a transition metal-free, economical, environmentally compatible, and highly efficient method for the production of complex heterocyclic moieties with thiazines as constituent parts [29]. They synthesized their complex 1,4-thiazine analogues (Scheme 15) by treating 4,5-dichloro-2-(tetrahydro-2*H*-pyran-2-yl)pyridazin-3(2*H*)-one with *N*-benzyl-2-mercaptoacetamide using Cs_2_CO_3_ as catalyst in DMF [29]. The one-pot Smiles rearrangement process is quite useful in organic chemistry [30] and it should be fully explored for the synthesis of other complex thiazine derivatives that are important from medicinal point of view.

**Scheme 16:**
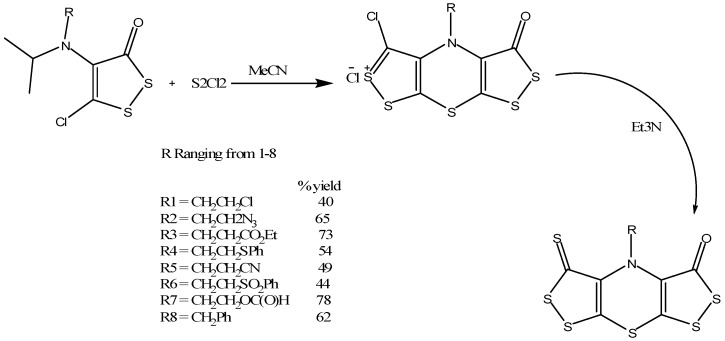
Synthesis of bis(1,2)dithiolo derivatives of 1,4-thiazine [31].

Konstantinova and coworkers synthesized the bis(1,2)dithiolo derivative of 1,4-thiazine class (Scheme 16) by treating 4-isopropylamino-5-chloro-1,2-dithiole-3-ones with sulfuryl chloride in acetonitrile as a solvent that resulted in the formation of an intermediate salt of bis(1,2)dithiolo(1,4)thiazine chloride [31,32]. When this intermediate salt is further treated with triethylamine (Scheme 16), it resulted in the desired product [31]. The product formed was further confirmed and analyzed by various techniques. These thiazine derivatives were also checked for their antiviral properties using the nucleocapsid protein of the feline immunodeficiency virus (FIV) [33] in vitro cell culture that showed activity in nanomolar concentration and low toxicity [34]. Thus these thiazine analogues may be suitable candidates for HIV treatment and could be tested further to check for potency in mice and human HIV infected tissues.

## 3. Biological Activities of Thiazine and Benzothiazine Derivatives

Heterocyclic thiazine and benzothiazine compounds are a crucial part of medicinal organic chemistry due to their useful medicinal properties. The largely unexplored heterocyclic compounds like thiazines possess a variety of pharmacological activities, ranging from antitumor, antipsychotic and anti-inflammatory properties on the one hand, while on the pathogenic side, they are equally important due to their antibacterial, antitubercular, antifungal, antiviral and antiprotozoal activities. Thus thiazine compounds display a wide range of beneficial properties [6]. These activities are discussed one by one in the following paragraphs.

### 3.1. Antimicrobial Activities

**Figure 2:**
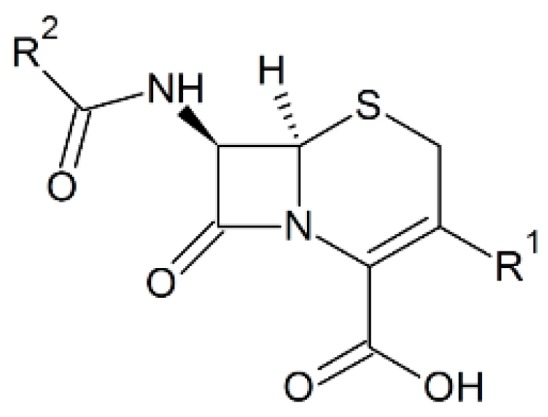
Chemical structure of the cephalosporin nucleus.

Phenothiazines and their novel derivatives exhibit valuable biological activities both in vivo and in vitro. These compounds show good results against various diseases caused by bacteria, viruses, fungi, mollusca, or protozoa. The activities of these compounds were examined by applying them on various organisms such as mammals infected with pathogenic bacteria and viruses, cell lines, etc. [35]. The 1,3-thiazine moiety is the functional part of cephalosporins that are the β-lactam antibiotics, active against most Gram positive and a number of Gram negative pathogenic bacteria [36]. Cephalosporins have a similar mode of action like penicillins and have an identical β-lactam ring, however, there are more atoms at the side rings as presented in Figure 2 [37]. Most of the penicillin-resistant bacteria are sensitive to cephalosporins, although there are some exceptions [37]. Bacterial resistance is a common problem to cephalosporins, for example the enteric bacteria are resistant to almost all of the third and fourth generation drugs [38]. There are several strains of *Staphylococcus aureus* that are even resistant to the fifth generation cephalosporins like ceftobiprole and ceftaroline [39]. Therefore, novel strategies are being devised to generate future generation cephalosporins [38]. In addition to broad spectrum activities, 1,3-thiazine and its derivatives contains wonderful properties like antitumor, insecticide, and fungicidal [19,20,40,41]. Further, these can be used as anti-radiation agents in radiation-sickness [19,20,40,41]. The 4-hydroxy-*N*′-(benzylidene)-2*H*-benzo(*e*)(1,2) thiazine-3-carbohydrazide 1,1-dioxides [10] compounds possess not only antibacterial but also has radical scavenging activities and these properties are raised when more lipophilic is the compound [14,15]. Thus, such thiazine derivatives are useful chemical tools in biochemistry research and can be used in respiratory and photosynthetic electron transfer research where the free radicals are produced during normal activity as well as malfunction of some pathways. Patel et al. made around forty different 1,2-benzothiazine derivatives, among which some only exhibited antibacterial activity against Gram positive bacteria [42].

A number of 1,4-thiazine analogues that were modified form of their parent thiazine compounds displayed broad spectrum antibacterial action in vitro. They were active against several types of both Gram positive and Gram negative pathogenic bacteria [43]. The imides and *N*-carboxymethyl imides based 2-substituted *N*-acylphenothiazines also have antimicrobial activity [44].

Thiazine compounds are also useful as reactants in the preparation of several other important classes of synthetic antibiotics like quinolones that are used for the treatment of several pathogenic bacterial diseases [45]. For example Parai and colleagues prepared several 1,4-thiazine analogues and (*S*)-3-methyl-1,4-benzoxazine [46]. These thiazine derivatives can be made using copper as a catalyst through the intramolecular *N*-aryl amination reaction mechanism on substituted 2-(2-bromo-phenylthio)ethanamines [47] that can be prepared by treating 2-bromobenzenethiol with Boc-protected amino alcohol compounds [46]. The (*S*)-3-methyl-1,4-benzoxazine can be used as a reactant for the synthesis of levofloxacin which is a common quinolone antibiotic [46]. There are several green synthesis methods in which thiazine derivatives and quinolones can be synthesized in the same reaction step [48,49].

Jeleń et al. explored the possible antimicrobial role of 44 other phenothiazine analogues that contained the quinobenzothiazine nucleus in their structure. Most of these phenothiazines exhibited different levels of inhibitory action on the growth of common pathogenic bacteria and fungi [50]. It is also important to mention that 6-(1-methyl-2-piperidylethyl) quinobenzothiazine was highly toxic for common pathogenic *Staphylococcus* and *E. coli* while a multi-substituted thiazine like 6-methane sulfonylaminobutyl-9-methylthioquinobenzothiazine was lethal for all the tested microbes [50]. The hybrid forms of pyrazine-1,3-thiazine derivatives displayed antiviral activities in micromolar concentration against HIV, influenza A virus, Enterovirus 71 and Coxsackievirus B3 [51]. These hybrid derivatives were active against HIV reverse transcriptase, influenza A neuraminidase enzyme in enzyme based assays [51]. The Enterovirus 71 and Coxsackievirus B3 infection was cured with the respective pyrazine-1,3-thiazine derivatives with high specificity [51]. Heavy metals with their ligands prepared using the Schiff base obtained from 5-acetyl-4-hydroxy-2*H*-1,3-thiazine-2,6(3*H*)-dione and 2-aminophenol have broad spectrum antibacterial and antifungal activities [52]. The heavy metals with thiazine compounds in different assays exhibit variable results of antibacterial and antifungal activities [52].

### 3.2. Antitubercular Activities

*Mycobacterium tuberculosis* is a highly resistant pathogenic bacterium which is almost resistant to a number of the drugs and there is always a requirement to develop new drugs to control the resistant pathogen challenges. Koketsu et al. synthesized around eight different 5,6-dihydro-4*H*-1,3-thiazine analogues and they were tested on mycobacterium species, where several of them have inhibitory activities [53]. Most of these compounds have high antitubercular activity and are effective in microgram concentration, thus 1,3-thiazine derivatives have potential antimycobacterial activities [53]. The ethyl 6-(4-chlorobenzoyl)-1,1-dioxo-3,5-diaryl derivatives of the 1,4-thiazinane-2-carboxylates are also highly active compound against different species of mycobacterium that are highly resistant to other drugs [54]. They can be obtained very easily by treating ethyl 2-[(2-oxo-2-arylethyl)sulfonyl]acetates with various branched aromatic aldehydes and amines and the reaction is catalyzed by l-proline [54]. The advantages of this method are that the products (1,4-thiazines) are obtained in good yield and it is a green synthesis method [54].

### 3.3. Antimalarial Activities

The search for thiazine-based antimalarial drugs are quite old and it dates back to 1891 when Guttmann and Ehrlich used thiazine dyes for the treatment of malaria [55]. The thiazine dyes are quite potent and selective for the inhibition of plasmodium growth [55]. In the past it was observed that the 2,4-diamino-1-(*p*-chlorophenyl)-1,6-dihydro-6,6-dimethyl derivative of 1,3,5-triazine [56] which is a chloroguanidine metabolite has some activity against plasmodium while the 3,5-dichlorophenyl analogue which is a metabolite of dichloroguanide, is more potent against plasmodium [57]. The Australian marine sponge *Plakortis lita* contained four thiazine-based alkaloids called thiaplakortones, among which thiaplakortone A is the most active against both chloroquine-sensitive and -resistant strains of *Plasmodium falciparum* with IC_50_ values in the range of 51 to 6.6 nM [58]. A total synthesis of thiaplakortone is also available and several different derivatives for drug resistant plasmodium species can now easily be synthesized through this established method [59]. A number of amide and urea containing groups of thiaplakortone has also been synthesized and checked for their antiplasmodial activity [60,61]. It was also observed that several of these analogues have IC_50_ values of less than 500 nM, with good aqueous solubility [60,61].

### 3.4. Antifungal Activities

Some thiazine have antifungal properties, for example Vicentini et al. synthesized pyrazole forms of 1,3-thiazine and 1,3,5-thiadiazines derivatives [62]. They tested them on rice blast caused by *Magnaporthe grisea* [63]. Both the thiazine derivatives inhibit the growth of the fungus when used in the concentration range of 10 to 200 μg∙mL^−1^ while in some cases, they can stop the growth of mycelium when used at concentration of 10 μg·mL^−1^. Thus, these thiazine derivatives have quite impressive mycotoxic activities [62,63]. The substituted forms of 1,4-benzothiazines and 1,5-benzothiazepines and their annelated derivatives are pharmacologically very important as some of them can be used as antimicrobials, antiviral, antibacterial and antifungal drugs [64]. The 1,4-thiazines are toxic to the two fungal species of *Aspergillus niger* and *Aspergillus fumigatus* [43].

### 3.5. Anticancer Activities

**Figure 3:**
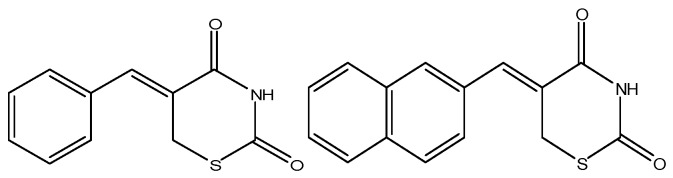
Chemical structure of bicyclic and tricyclic thiazinediones that have anticancer activity [65].

The thiazinediones (Figure 3), prepared by Ferreira and colleagues showed anticancer activity against leukemia cells [65]. It was observed that the DNA fragmentation (cell death) was produced by various aromatic groups attached to the 1,3-thiazine-2,4-diones that possibly intercalate with the targeted DNA resulting in DNA fragmentation and ultimate cell death [65]. It was also proposed that the caspase cascade activation occurs, along with the disparity in intracellular calcium ion, disturbance in basic cell functional organelles [65,66]. The newly synthesized compound 2-(2,4-dihydroxyphenyl)thieno-1,3-thiazin-4-one (BChTT) can be used to stop lung, colon and other types of progressing cancers and it is non-toxic to normal body cells and organs [66]. These thiazine derivatives stop the synthesis of DNA and ceased the different cell growth stages by decreasing the activity of p38 kinase and cyclin D1 enzymes [66].

**Figure 4:**
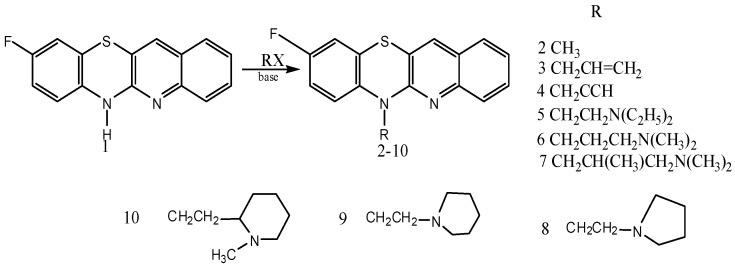
Synthesis of tetracyclic azaphenothiazine derivatives.

Jeleń and coworkers have prepared novel tetracyclic azaphenothiazines from 6-substituted 9-fluoroquinobenzothiazines (Figure 4) which showed inhibition of different types of blood cancer cells and are also having antitumor, antiproliferative and anti-inflammatory properties. Substitution at the thiazine nitrogen of 9-fluoroquinobenzothiazine decreased its toxicity [67]. Some of the thiazine derivatives are close to cisplatin for cancer and cyclosporine A for anti-inflammation in terms of activity [67]. Thus the promising low cost of thiazine based anticancer and anti-inflammatory drugs should be considered and further trials need to be done on them so that they can come early to the market and we can control the plight of cancer in our society.

Ha et al. reported a skin depigmenting agent made from synthetic 1,3-thiazine compounds [68]. These thiazine analogues inhibit the activity of tyrosinase enzymes in different kinds of cells and blocking the melanin biosynthesis pathway, thus they possess anti-melanogenesis effects [68]. The 1,2-thiazine 1-oxides are very useful for the synthesis of several different conjugated forms of pyrrole and benzodiazepines or benzothiadiazepines that have antitumor properties [69,70]. The monoterpene-fused 2-imino-1,3-thiazines also exhibit anticancer properties and they can be made in a few steps using green synthesis methods [71]. A combination of ruthenium II-arene fragments with oxicams showed that they are useful in the treatment of colon, breast cancers and carcinogenesis-based inflammations [72].

### 3.6. Anti-Inflammatory Activities

**Figure 5:**
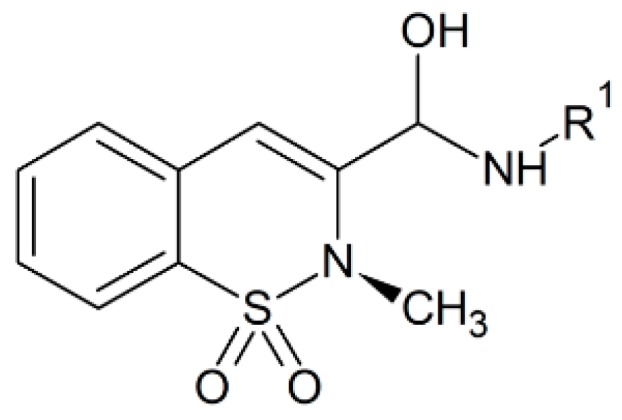
Chemical structure of the oxicam nucleus.

The thiazine alkaloids present in the New Zealand *Aplidium* ascidian species have anti-inflammatory activity as they have proved to inhibit neutrophil-based superoxide production [73]. Chia and colleagues made different thiazine analogues conjugated with quinolones and quinones that suppress the free radical production by neutrophils in vitro when used in small concentrations. Thus, they can be future NSAID alternatives [74]. Several thiazine derivatives mentioned above have both anti-inflammatory and analgesic activities at lower doses and are also safe at lower doses in gastrointestinal irritation [23]. Derivatives of 2-arylimino-5,6-dihydro-4*H*-1,3-thiazine act as cannabinoid receptor agonists for CB1 and CB2 receptors and were found to have analgesic activity. In these medicinal compounds the oxazine moiety has been merged as an isosteric alternative for the thiazine ring of actual compound and one of the compounds possess oral bioavailability as well as analgesic properties [75,76,77]. The benzothiazepines conjugated with other thiazines were tested on neuronal receptors, where only one derivative showed inhibitory effects on the central nervous system while most of the others exhibited anti-inflammatory properties [78]. Benzothiazines are quite bioactive and this activity, mostly depends on the attached side groups [79,80]. The current oxicam medicines are chemically benzothiazines in nature (Figure 5) and they are second generation non-steroidal drugs that act as an anti-inflammatory agent [79,80]. The current medicinal use of oxicams is restricted due to their adverse effects, although a number of research efforts were done to increase their potency against inflammatory conditions with lower side effects [79,80]. Xu et al. resolved the crystal structure of cyclooxygenases in binding mode with oxicam compounds at 2–2.45 Å resolution [81,82]. The oxicam compounds bind inside the active site of cyclooxygenases through two water-mediated hydrogen bonds and this mode of interaction is different from that of other NSAIDs [81,82]. There is a hydrogen bonding interaction between the 4-OH group of thiazine and serine-530 of the cyclooxygenase while the heteroatom of carboxamide ring interacts with tyrosine-385 and serine-530 via water-mediated hydrogen bonding interaction [81]. The second water-based H-bond is formed between the nitrogen atom of thiazine and oxygen atom of carboxamide to the arginine-120 and tyrosine-355 [81]. The oxicams also possess free radical scavenging properties [83,84,85,86]. Eleftheriou et al. reported that imbalanced cyclooxygenase-1 and 2 functions resulted in inflammation and these enzymes can be blocked with benzo/benzisothiazolimino-5-aryliden-4-thiazolidinones using a balanced selection of several enzymes involved in inflammation mechanism [87]. The benzothiazolyl basic unit with other groups attached can be explored further as active inhibitors for the whole cyclooxygenase enzyme family [87]. They can provide a scaffold on which full fragment-based drug ligands (FBDL) as inhibitors can be made for different enzymes [87]. In this report a chemoinformatics method was used and a fragment library of the above mentioned drugs were created [87]. These fragments were docked with the cyclooxygenases and along with this computation study; these drugs were also tested both in vitro and in vivo in biological samples [87]. These fragment based ligands can also be used as possible covalent inhibitors and should be studied on different enzymes involved in various diseases.

### 3.7. Antiarrhythmic Activities

Some thiazine derivatives are also useful for the treatment of heart diseases, for example Galanski and coworkers made several position 6-substituents of 1,4-thiazine derivatives like 6-ethyl and 6-benzylthieno(2,3-*b*)(1,4)thiazine analogues that relax the vascular smooth muscle and terminal ileum [88,89]. They observed that the thieno(3,2-*b*)(1,4)thiazines [88] are better vasopressin antagonists as compared to the thieno(2,3-*b*)derivatives of the 1,4-thiazine and 1,4-thiazepine compounds [88]. The same compounds were further analyzed as non-peptide vasopressin antagonists on different heart muscles and it was concluded that they are useful for the cure of cardiac arrest due to congestion of valves, hypertension or peripheral vascular diseases and renal malfunctions [88,89].

### 3.8. Antidiabetic Activities

Aldose reductase is considered to be one of the key enzymes involved in diabetic complications [90,91,92,93] and that is why it is a suitable drug target for diabetic treatment [94]. For this purpose some 1,2-benzothiazine 1,1-dioxide acetic acid derivatives were synthesized and tested against aldose reductase, among them the double halogenated benzene analogues showed the most promising inhibition capacity [94,95]. The structure activity relationship and molecular docking of these compounds with aldose reductase showed that saturated carboxylic acid have a higher binding affinity for the enzyme as compared to the unsaturated carboxylic acid [94,95].

### 3.9. Anti-Alzheimer Activities

Recently it is also discovered that the bicyclic thiazines can be used for controlling Alzheimer’s disease (AD). The bicyclic thiazine targets the beta-secretase 1 (BACE1) enzyme which is an important target protein in the fight against this disease [96]. This work is still in progress in designing more effective thiazine derivatives that can bind strongly in different pockets of the BACE1 enzyme. The spiro-analogues of 1,3-thiazine that are potential neuroprotectors have the ability to inhibit the amino acid-induced calcium ion uptake into rats’ brain cortex special functional parts like synaptosomes. The blocking activity of such drugs is due to the specified chemical structure of the side chain attached to the exocyclic nitrogen atoms [97,98]. Another useful activity of thiazine in AD is that the Thiazine Red^+^ dye is used for the identification of Alzheimer affected areas in the rat brain in laboratories [99].

It was also recorded that the 2,6-dicarbethoxy-3,5-diaryltetrahydro-1,4-thiazine-1,1-dioxide analogues work better in maximal electroshock, subcutaneous pentylenetetrazole methods for finding anticonvulsant properties [10]. The experimental evidence proved their anticonvulsant properties that are mostly attributed to the kind and position of side chain present on the phenyl ring [10]. It is also an advantage that some structurally close compounds had no toxicity in the neurotoxic experiments [12].

Beside the above mentioned bioactivity of thiazine against various diseases, other active chemical compounds can also be synthesized from them that have medicinal importance in the fight against different types of viral and cancerous diseases.

## 4. Conclusions and Future Perspectives

The available data in the literature reveal that thiazines are a considerably vital group of heterocyclic compounds and their applications in the treatment of different infectious and inflammatory diseases are promising. This review summarized the design and synthesis of different thiazine derivatives by various methods, including microwave irradiation, solvent free reactions, one pot synthesis and other environmentally friendly methods which should be adapted easily in different biomedical and pharmaceutical research. Moreover, the study of thiazine has attracted the interest of medicinal chemists and biochemists up to a great extent and showed that this potent molecule possess capabilities for designing potential bioactive agents. This review provides a preliminary information and its references will greatly help medicinal chemists in making better and highly efficient, cost effective and easily available thiazine derivatives. Further, we can conclude that many other derivatives of thiazine, their nanoparticles as well as their complexes with transition metals can be synthesized that may possess effective pharmacological activities.

## Abbreviations

IR: Infra-red spectroscopy
EMIS: Electromagnetic induction spectroscopy
CAN: Ceric ammonium nitrate
CB: Cannabinoid receptors
AVP: Arginine vasopressin
PBTDs: Pyrrolobenzothiadiazepines
ALR2: Aldose reductase 2
NSAIDs: Nonsteroidal anti-inflammatory drugs
FBDL: Fragment based drug ligands
AD: Alzheimer’s disease
BACE1: beta-secretase 1
